# Haemoglobin Mass and Running Time Trial Performance after Recombinant Human Erythropoietin Administration in Trained Men

**DOI:** 10.1371/journal.pone.0056151

**Published:** 2013-02-13

**Authors:** Jérôme Durussel, Evangelia Daskalaki, Martin Anderson, Tushar Chatterji, Diresibachew H. Wondimu, Neal Padmanabhan, Rajan K. Patel, John D. McClure, Yannis P. Pitsiladis

**Affiliations:** 1 Institute of Cardiovascular and Medical Sciences, College of Medical, Veterinary and Life Sciences, University of Glasgow, Glasgow, United Kingdom; 2 Strathclyde Institute of Pharmacy and Biomedical Sciences, University of Strathclyde, Glasgow, United Kingdom; 3 Department of Medical Physiology, Addis Ababa University, Addis Ababa, Ethiopia; Johns Hopkins School of Medicine, United States of America

## Abstract

Recombinant human erythropoietin (rHuEpo) increases haemoglobin mass (Hb_mass_) and maximal oxygen uptake ( O_2 max_).

**Purpose:**

This study defined the time course of changes in Hb_mass_, 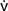 O_2 max_ as well as running time trial performance following 4 weeks of rHuEpo administration to determine whether the laboratory observations would translate into actual improvements in running performance in the field.

**Methods:**

19 trained men received rHuEpo injections of 50 IU•kg^−1^ body mass every two days for 4 weeks. Hb_mass_ was determined weekly using the optimized carbon monoxide rebreathing method until 4 weeks after administration. 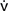 O_2 max_ and 3,000 m time trial performance were measured pre, post administration and at the end of the study.

**Results:**

Relative to baseline, running performance significantly improved by ∼6% after administration (10∶30±1∶07 min:sec vs. 11∶08±1∶15 min:sec, p<0.001) and remained significantly enhanced by ∼3% 4 weeks after administration (10∶46±1∶13 min:sec, p<0.001), while 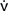 O_2 max_ was also significantly increased post administration (60.7±5.8 mL•min^−1^•kg^−1^ vs. 56.0±6.2 mL•min^−1^•kg^−1^, p<0.001) and remained significantly increased 4 weeks after rHuEpo (58.0±5.6 mL•min^−1^•kg^−1^, p = 0.021). Hb_mass_ was significantly increased at the end of administration compared to baseline (15.2±1.5 g•kg^−1^ vs. 12.7±1.2 g•kg^−1^, p<0.001). The rate of decrease in Hb_mass_ toward baseline values post rHuEpo was similar to that of the increase during administration (−0.53 g•kg^−1^•wk^−1^, 95% confidence interval (CI) (−0.68, −0.38) vs. 0.54 g•kg^−1•^wk^−1^, CI (0.46, 0.63)) but Hb_mass_ was still significantly elevated 4 weeks after administration compared to baseline (13.7±1.1 g•kg^−1^, p<0.001).

**Conclusion:**

Running performance was improved following 4 weeks of rHuEpo and remained elevated 4 weeks after administration compared to baseline. These field performance effects coincided with rHuEpo-induced elevated 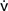 O_2 max_ and Hb_mass_.

## Introduction

Erythropoietin is a glycoprotein hormone produced primarily in the kidney that regulates red blood cell mass by stimulating the survival, proliferation and differentiation of erythrocytic progenitors [1]. In healthy subjects, administration of recombinant human erythropoietin (rHuEpo) increases haemoglobin concentration not only by the well known increase in red blood cell mass but also by a decrease in plasma volume [2], [3], [4]. The decrease in plasma volume, which precedes the increase in red cell mass, appears to be a rapid responding mechanism regulated by the renin-angiotensin-aldosterone system to control haematocrit [2], [3]. Theoretically, normal human red blood cells can persist in the circulation for approximately 17 weeks [5]. However, neocytolysis, the selective hemolysis of young circulating red blood cells which contributes to the regulation of red cell mass, seems to appear during specific conditions that cause a rapid decrease in erythropoietin levels such as spaceflight, high altitude exposure or blood doping [5], [6], [7], [8], [9]. Little is known about the time course of these mechanisms post rHuEpo administrations. In this study, heamoglobin mass (Hb_mass_) and related blood volumes were repeatedly determined using the optimized carbon monoxide (CO) rebreathing method [10], [11] pre, during and post rHuEpo administration in healthy trained men.

Most studies investigating the effects of rHuEpo on exercise performance have used maximal oxygen uptake (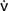 O_2 max_) tests. However, 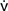 O_2 max_ tests alone may be of little help in predicting exercise performance among athletes of similar ability [12], [13], [14], [15]. One study assessed submaximal performance in healthy male subjects and reported improvements of more than 50% in time to exhaustion on a cycle ergometer at a given 80% of 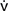 O_2 max_ whereas the increase in 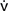 O_2 max_ was approximately 12% [16]. Despite being a very interesting finding which questions the mechanisms by which rHuEpo improves submaximal exercise performance [17], the validity of the time to exhaustion test in assessing human exercise performance has been questioned [18]. In addition to being more variable than time trial tests [19], time to exhaustion tests are less likely to mimic the exercise performance environment [18], [19]. There are indeed only minimal occasions where an athlete is required to maintain a constant intensity until volitional exhaustion compared with self-paced exercise [18], [19].

The main purpose of the present study was to investigate the time course of Hb_mass_ and related blood parameters as well as changes in running 3,000 m time trial performance following 4 weeks of rHuEpo administration in healthy trained men in order to determine whether the augmented 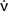 O_2 max_ observed in laboratory based experiments would translate into actual improvements in running performance in the field.

## Methods

### Ethics Statement

This study was approved by University of Glasgow Ethics Committee and conformed to the Declaration of Helsinki. All subjects underwent a medical assessment and provided written informed consent to participate. This study was part of a larger research project funded by the World Anti-Doping Agency (WADA).

### Subjects

Nineteen healthy non-smoking trained men (mean ± SD, age: 26.0±4.5 yr, body mass: 74.8±7.9 kg, height: 179.8±5.4 cm) participated in this study. The subjects were regularly engaged in predominantly endurance-based activities such as running, cycling, swimming, triathlon and team sports. The subjects were divided into two groups for exercise performance analysis. The first group included subjects who had a history of running (n = 10) and the second group included the remaining subjects who were involved in other sporting activities (n = 9). Subjects were requested to maintain their normal training but abstain from official sporting competition for the duration of the research protocol. One of the subjects in the running group suffered from nasal obstruction and coughing during the study and could not perform the time trials post rHuEpo administration. The running performance data of this subject was excluded from the analyses.

### Experimental Design

Each subject subcutaneously self-injected 50 IU•kg^−1^ body mass of rHuEpo (NeoRecormon, Roche, Welwyn Garden City, UK) every second day for 4 weeks. Daily oral iron supplementation (∼ 100 mg of elemental iron, Ferrous Sulphate Tablets, Almus, Barnstaple, UK) was given during the 4 weeks of rHuEpo administration. Venous blood samples from an antecubital vein were obtained in triplicate at baseline (over 2 weeks prior to the first rHuEpo administration), during rHuEpo administration (on days 2, 4, 8, 10, 14, 16, 20, 22, 26, 28) and for 4 weeks after rHuEpo administration (on days 30, 35, 41, 43, 48, 50, 57). All blood samples were taken after 10 min of rest in the supine position [20]. Blood samples were homogenized using a roller mixer and then measured in triplicate using a Sysmex XT-2000i (Sysmex UK, Milton Keynes, UK). The mean value of the triplicate was reported. Resting blood pressure and heart rate were recorded three times on both arms in the supine position before blood sampling using an automated cuff oscillometric device (Boso-Medicus, Bosch & Sohn GmbH, Jungingen, Germany).

### Measurements of Haemoglobin Mass and Related Blood Volumes

Hb_mass_ was determined in triplicate prior to the first rHuEpo injection and then weekly up to 4 weeks post rHuEpo administration using the optimized CO-rebreathing method as previously described [10], [11]. Briefly, a bolus of chemically pure CO dose of 1.0 mL•kg^−1^ body mass was administered with the first breath through a spirometer and rebreathed for 2 min with 4 L of oxygen. Change in percent carboxyhaemoglobin in venous blood samples from baseline to 8 min after CO administration was measured using a blood gas analyzer (ABL 725, Radiometer, Copenhagen, Denmark). CO concentration was measured using the Pac 7000 Draeger CO-analyzer (Draeger Safety, Northumberland, UK) and an estimated alveolar ventilation of 7.5 l•min^−1^ was used for calculations. Hb_mass_ as well as blood, red cell and plasma volume were calculated as previously described elsewhere [11]. Blood volume was derived by dividing Hb_mass_ by the haemoglobin concentration. Red cell volume was obtained by multiplying blood volume by the haematocrit and plasma volume was then calculated by subtracting red cell volume from blood volume. The typical error of measurement for Hb_mass_ calculated from the two first baseline measurements was 1.5% (95% confidence interval (CI) 1.1 to 2.2%). The typical error of measurement for blood, red cell and plasma volume between the two weeks prior to the first rHuEpo administration were 2.4%, 1.6% and 3.6% (CI 1.8 to 3.5, 1.2 to 2.4 and 2.7 to 5.3), respectively.

### Running Performance Assessment

Two 3,000 m time trials separated by at least one day rest were performed on a 200 m indoor athletic track (Kelvin Hall, Glasgow, UK) pre, post rHuEpo administration and at the end of the study. Verbal encouragement was given with feedback provided for the split time and remaining laps. The best performance on each occasion was used for analysis. The typical error of measurement for time trial performance calculated from the two tests on each phase was 1.3% (CI 1.1 to 1.7). Borg’s rating of perceived exertion (RPE) was recorded at the completion of the time trial. Temperature and humidity were recorded using a hygrometer. In addition to the time trial, 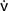 O_2 max_ was determined pre, post rHuEpo administration and at the end of the study using an incremental test to exhaustion on a motorised treadmill. Both continuous or discontinuous protocols were used because of other purposes of the study (the speed was increased by 1 km•h^−1^•min^−1^ or by 2 km•h^−1^ every 3 min with 3 min of active recovery at a walking pace between each bout, respectively). Following a 30 min recovery, 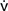 O_2 max_ was verified using a square-wave protocol to exhaustion at a speed equivalent to the end speed attained during the incremental test minus 1 km•h^−1^ [21], [22]. Gas exchange was measured breath by breath using an automated metabolic gas analysis system (Cosmed Quark b2, Cosmed, Rome, Italy).

### Statistical Analysis

Individual mean value was calculated when more than one blood sample was collected per week before further analysis. Changes over time in Hb_mass_, blood, red cell and plasma volume were assessed using t-test to determine whether the slope of these parameters was significantly different from zero. Time trial performance, 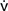 O_2 max_ and blood parameters data for the key stages of the study were analyzed using repeated measures ANOVA. Relationships between time trial performance, 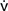 O_2 max_ and Hb_mass_ were assessed using Pearson’s product moment correlation coefficients.

## Results

### Running Performance (Table 1)

Running performance data of both groups combined (n = 18) are presented in this paragraph (Table 1 for separate groups). Irrespective of running history, time trial performance significantly improved by ∼6% post administration (10∶30±1∶07 min:sec vs. 11∶08±1∶15 min:sec, p<0.001) and remained significantly enhanced by ∼3% 4 weeks after rHuEpo compared to baseline (10∶46±1∶13 min:sec vs. 11∶08±1∶15 min:sec, p<0.001). RPE did not significantly differ between the time trials (p>0.05). Temperature (19.4±2.8°C) and humidity (46.6±9.0%) remained relatively constant (p>0.05). Relative to baseline, 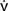 O_2 max_ significantly increased post administration (60.7±5.8 mL•min^−1^•kg^−1^ vs. 56.0±6.2 mL•min^−1^•kg^−1^, p<0.001) and remained significantly increased 4 weeks after rHuEpo (58.0±5.6 mL•min^−1^•kg^−1^ vs. 56.0±6.2 mL•min^−1^•kg^−1^, p = 0.021). 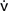 O_2 max_ was at least moderately correlated with Hb_mass_ as well as time trial performance throughout the study (range, r = 0.48 to r = 0.88, p<0.05). However, a significant correlation in individual responses compared to baseline between parameter was only observed for Hb_mass_ and time trial performance at the end of the study (r = −0.68, p = 0.002).

**Table 1:** Running 3,000 m time trial performance and maximal oxygen uptake.

	Group 1			Group 2		
	Baseline	End ofrHuEpo	End of the study	Baseline	End ofrHuEpo	End of the study
3,000 m total time (min:sec)	10∶12 (0∶42)	9∶40* (0∶37)	9∶53* (0∶43)	12∶05 (0∶55)	11∶19* (0∶53)	11∶39* (0∶58)
1^st^ 1,000 m split (min:sec)	3∶16 (0∶13)	3∶10* (0∶12)	3∶13 (0∶13)	3∶53 (0∶27)	3∶36* (0∶14)	3∶41 (0∶20)
2^nd^ 1,000 m split (min:sec)	3∶26^†^ (0∶16)	3∶15*^†^ (0∶14)	3∶20^†^ (0∶14)	4∶05 (0∶17)	3∶54^†^ (0∶26)	3∶59^†^ (0∶20)
3^rd^ 1,000 m split (min:sec)	3∶29^†^ (0∶15)	3∶15* (0∶13)	3∶20* (0∶16)	4∶07 (0∶16)	3∶49*^†^ (0∶15)	3∶59^†^ (0∶19)
RPE scale (6–20)	18.0 (1.7)	18.4 (0.9)	19.0 (1.2)	17.7 (2.2)	18.4 (1.6)	18.6 (1.4)
O_2 max_ (mL•min^−1^•kg^−1^)	60.3 (5.0)	64.4* (3.9)	61.8 (3.4)	51.6 (3.5)	57.0* (5.1)	54.2* (4.7)

Group 1 included subjects who had a history of running (n = 9) and group 2 included the other subjects who were involved in other activities (n = 9). Values are means (SD). Significant differences compared to baseline values are indicated by *P<0.05. Significant differences compared to the first 1,000 m split time within the same time trial. ^†^ *P*<0.05. RPE: Borg’s rating of perceived exertion; O_2 max_: Maximal oxygen uptake.

### Haematological Parameters and Resting Blood Pressure and Heart Rate (Tables 2 and 3)

Table 2 illustrates the changes in the main haematological parameters for five key stages of the study. Relative to baseline values, both haematocrit and haemoglobin concentration gradually increased throughout the rHuEpo administration to reach a maximum approximately one week after the last injection (p<0.001) and remained significantly elevated 4 weeks post rHuEpo administration (p<0.001). Reticulocyte percent increased rapidly after the first two weeks of injections (p<0.001). Nadir with a very low inter-subject variation was reached approximately two weeks after injections ceased, which was significantly lower compared with baseline values (p<0.001). Body mass did not change during the study. rHuEpo administration did not induce any significant changes in resting systolic and diastolic blood pressure as well as in resting heart rate.

**Table 2:** Haematocrit, haemoglobin concentration, reticulocytes, haemoglobin mass, blood volumes, carboxyhaemoglobin and body mass before, during and 4 weeks post rHuEpo administration.

	Baseline	Week 2 of rHuEpo	End of rHuEpo	Week 2 postrHuEpo	Week 4 postrHuEpo
Haematocrit (%)	41.9 (1.8)	44.7 (2.0)*	49.2 (2.0)*	47.7 (2.2)*	45.1 (1.7)*
Haemoglobin (g•dl^−1^)	14.4 (0.7)	15.2 (0.7)*	16.7 (0.9)*	16.1 (0.9)*	15.6 (0.7)*
Reticulocytes (%)	1.07 (0.31)	2.57 (0.44)*	1.46 (0.41)*	0.44 (0.13)*	0.55 (0.15)*
Hb_mass_ (g•kg^−1^)	12.7 (1.2)	13.4 (1.3)*	15.2 (1.5)*	15.1 (1.4)*	13.7 (1.1)*
Hb_mass_ (g)	947 (109)	1001 (127)*	1131 (131)*	1126 (136)*	1023 (132)*
Blood volume (L)	6.6 (0.9)	6.6 (1.0)	6.8 (0.8)	6.7 (0.9)	6.6 (0.8)
Red cell volume (L)	2.8 (0.3)	2.9 (0.3)*	3.3 (0.4)*	3.3 (0.4)*	3.0 (0.4)*
Plasma volume (L)	3.8 (0.6)	3.7 (0.7)	3.5 (0.4)*	3.4 (0.6)*	3.6 (0.5)
Carboxyhaemoglobin (%)	0.73 (0.15)	0.77 (0.16)	1.04 (0.19)*	1.01 (0.20)*	0.65 (0.15)
Body mass (kg)	75.1 (8.4)	74.7 (7.8)	75.0 (7.9)	74.6 (7.6)	74.6 (7.5)

*N* = 19. Values are means (SD). Significant differences compared to baseline values are indicated by *P<0.05. Hb_mass_: Haemoglobin mass.

### Haemoglobin Mass and Blood Volumes (Tables 2, 4 and Fig. 1)

**Figure 1:**
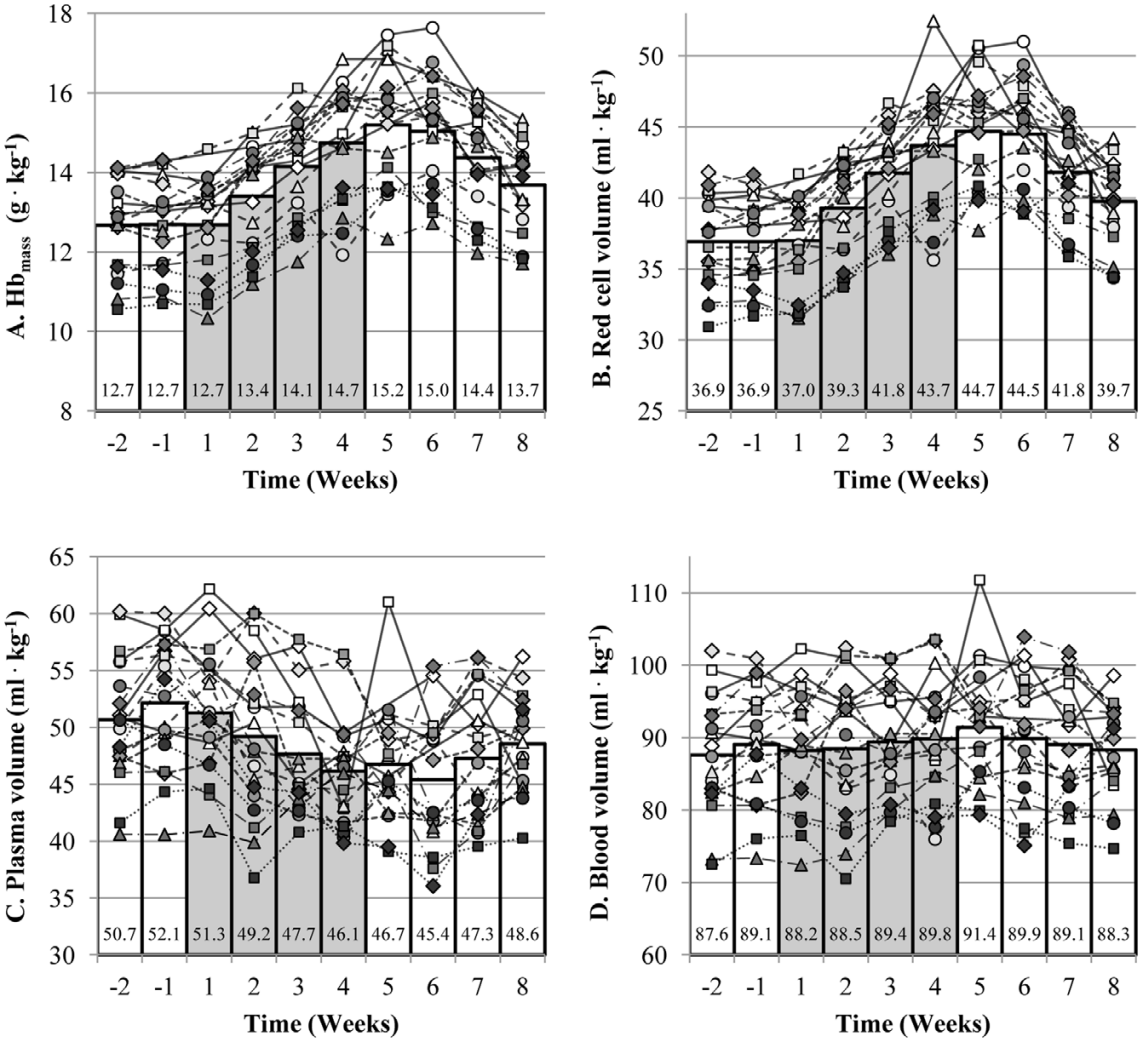
Individual changes in Hb_mass_ (A), red cell (B), plasma (C) and blood (D) volume. Each line corresponds to one subject and each symbol corresponds to the same subject in all figures. Individual mean value was calculated when more than one blood sample was collected per week. The bar graphs represent the mean values of the 19 subjects. The grey bars represent the 4 weeks of rHuEpo administration.

Relative to baseline, Hb_mass_ and plasma volume were significantly increased (p<0.001) and decreased (p = 0.004) at the end of the rHuEpo administration, respectively. Hb_mass_ and red cell volume gradually increased by approximately 40 g•wk^−1^ (p<0.001) and 135 mL•wk^−1^ (p<0.001), respectively (Fig. 1A and 1B). Plasma volume decreased significantly by approximately 100 mL•wk^−1^ (p<0.001) (Fig. 1C) while blood volume remained relatively unchanged (p = 0.32) (Fig. 1D). From week 1 to week 4 post rHuEpo administration, the rate of decrease in Hb_mass_ and red cell volume toward baseline values was similar to that of the increase during administration (Fig. 1A and 1B) but both Hb_mass_ and red cell volume were still significantly elevated 4 weeks post administration compared to baseline (p<0.001). Plasma volume was restored to pre-injection levels 4 weeks post administration (p = 0.108) (Fig. 1C).

**Table 3:** Resting blood pressure and heart rate before, during and 4 weeks post rHuEpo administration.

	Baseline	Week 2 ofrHuEpo	End ofrHuEpo	Week 2 post rHuEpo	Week 4 post rHuEpo
Systolic blood pressure (mmHg)	128 (9)	126 (7)	126 (6)	126 (7)	124 (7)
Diastolic blood pressure (mmHg)	70 (8)	69 (8)	69 (7)	70 (7)	69 (8)
Heart rate (beats•min^−1^)	62 (8)	60 (7)	60 (7)	59 (7)	61 (8)

*N* = 19. Values are means (SD). No significant differences compared to baseline values were observed.

**Table 4:** Changes in haemoglobin mass, blood, red cell and plasma volume during and for 4 weeks post rHuEpo administration.

	rHuEpo	Post rHuEpo
Haemoglobin mass (g•kg^−1^•wk^−1^)	0.54 (0.46 to 0.63)*	−0.53 (−0.68 to −0.38)*
Blood volume (mL•kg^−1^•wk^−1^)	0.34 (−0.36 to 1.05)	−0.60 (−1.20 to 0.00)
Red cell volume (mL•kg^−1^•wk^−1^)	1.80 (1.50 to 2.10)*	−1.77 (−2.22 to −1.33)*
Plasma volume (mL•kg^−1^•wk^−1^)	−1.30 (−1.94 to −0.66)*	0.50 (0.10 to 0.90)*

*N* = 19. Values are means (95% confidence interval). Significant changes are indicated by *P<0.05.

### Carboxyhaemoglobin (Table 2 and Fig. 2)

Relative to baseline values, basal percentage of carboxyhaemoglobin prior to the CO-rebreathing procedure was significantly elevated at the end of the rHuEpo administration (p<0.001). Percentage of carboxyhaemoglobin returned to pre-injection values 4 weeks post rHuEpo administration.

## Discussion

The aim of this study was to investigate the time course of Hb_mass_ and related blood parameters as well as changes in running 3,000 m time trial performance following 4 weeks of rHuEpo administration in order to determine whether the augmented 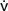 O_2 max_ would translate into actual improvements in running performance in the field. The main findings of this study are that, relative to baseline, running performance was significantly improved following 4 weeks of rHuEpo administration and remained significantly elevated 4 weeks post administration and that these performance effects coincided with significantly rHuEpo-induced elevated 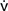 O_2 max_ and Hb_mass_.

### Running Performance

The effects of rHuEpo on exercise performance have principally been investigated using 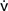 O_2 max_ tests. Despite slight differences in the frequency of injections and in the dosage used, rHuEpo administration for 4 to 6 weeks in healthy subjects has been shown to increase 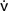 O_2 max_ by approximately 8%, which was confirmed by our observations [23], [24], [25], [26], [27], [28], [29]. However, 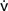 O_2 max_ considerably varies among professional athletes [30], [31] and 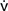 O_2 max_ tests alone may be of little help in determining and ranking exercise performance when athletes of similar endurance ability are compared [12], [13], [14], [15]. Time trial performance more closely reproduces competition conditions and more importantly allows the subjects to choose their own pace. It has previously been observed that blood doping via one unit of autologous blood transfusion, which increased the haematocrit by 5%, improved 10,000 m running performance by approximately one minute in 6 highly trained male distance runners, which corresponded to a ∼3% improvement [32]. However, to our knowledge, no study has measured running time trial performance in order to determine whether the augmented 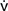 O_2 max_ after rHuEpo administration would translate into actual improvements in performance in the field.

**Figure 2:**
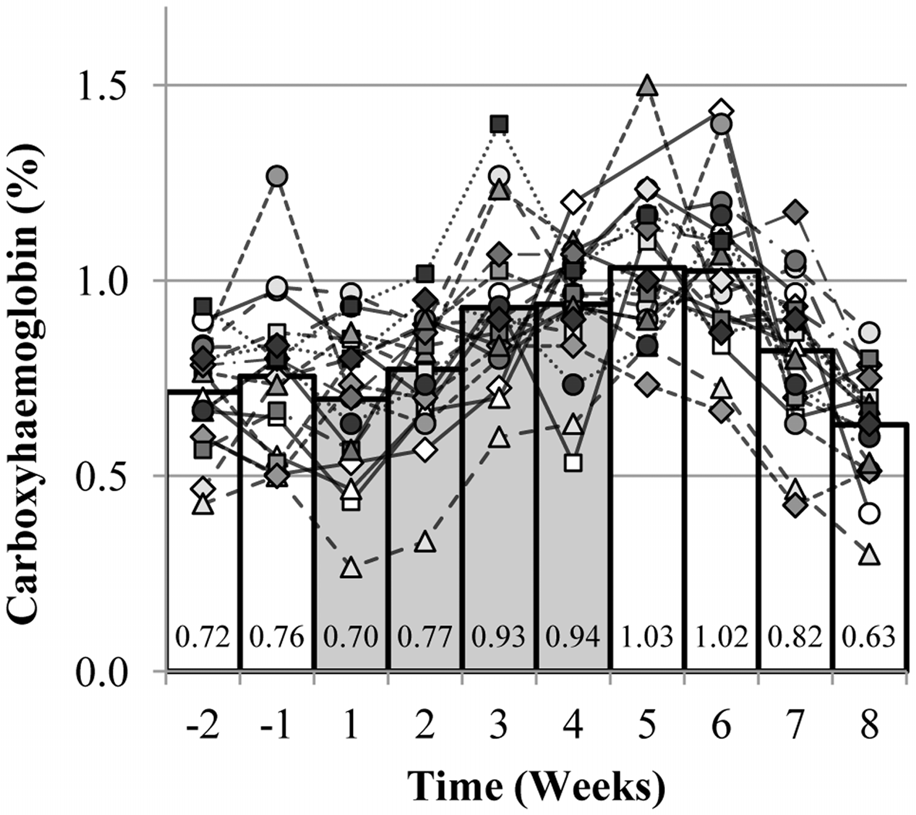
Individual changes in carboxyhaemoglobin. Each line corresponds to one subject. Individual mean value was calculated when more than one blood sample was collected per week. The bar graphs represent the mean values of the 19 subjects. The grey bars represent the 4 weeks of rHuEpo administration.

Relative to baseline, running performance improved post rHuEpo administration and remained enhanced 4 weeks after administration by approximately 6% and 3%, respectively. Following rHuEpo administration, subjects were able to maintain a faster pace throughout the 3,000 m time trial compared to baseline. While the augmented oxygen transport capacity illustrated by the increase in Hb_mass_ can explain the increase in 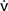 O_2 max_ post rHuEpo administration, it cannot really explain the significant improvement in running performance. Indeed, as oxygen supply and demand are adequately matched during submaximal exercise in healthy men, the rHuEpo-induced augmented oxygen transport capacity cannot be the reason of the improvement in time trial running performance observed in our study [12], [17]. In addition, failure to observe a strong correlation between individual changes in 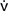 O_2 max_, Hb_mass_ and running performance may imply that rHuEpo administration improves submaximal exercise performance by other mechanisms, although it could also be partly explained by the limited sensitivity of the methods of measurement and by the limitations of the study (see next paragraph). Other mechanisms than the increase in oxygen transport capacity have been proposed to explain the effects of rHuEpo on exercise performance [33], [34]. For instance, it has been demonstrated that self-reported mood, cognitive function and perceived physical condition were improved following rHuEpo administration [35], [36]. rHuEpo may indeed exert effects in nonhematopoietic tissues including the brain [37], [38], [39]. Admittedly, two different studies conducted by the same research group did not reveal any measurable nonhematopoietic ergogenic effect of rHuEpo on exercise performance and the authors therefore concluded that the increased oxygen carrying capacity is the main if not the only reason why rHuEpo enhances exercise performance in healthy men [40], [41]. On the other hand, the central governor model can give an alternative view [12]. As the perception of effort of the subjects did not change and because the subjects were not limited by an inadequate oxygen supply neither before nor after administration as they ran at submaximal levels, the central governor model would argue that the subjects ran faster after rHuEpo administration because of greater recruitment of motor units allowed by a control mechanism of feed-forward and feedback to the brain [12; especially Fig. 2 of that paper].

This study was part of a larger research project funded by WADA and was designed primarily for other purposes. The present study was not blinded, did not include a control group and the subjects, yet involved in endurance activities, were not all runners. As such, we were unable to adequately assess changes in the training load during the study due to the absence of a placebo group and the heterogeneity of sporting/training activity of the subjects. It cannot totally be excluded that the performance enhancements observed may, therefore, be partly due to placebo effect, to an increase in the training load and to an improvement in pacing strategy especially for the non-runners. It was however reassuring that the typical error of measurement for the two time trials conducted on each phase was low and that the amplitude of the improvement observed was well above the confidence interval.

### Haemoglobin Mass and Haematological Parameters

The level of Hb_mass_ prior to the intervention reflected the regular participation in endurance activities of the subjects [42]. Following the first week of rHuEpo administration, Hb_mass_ gradually increased and reached its maximum one week after the last injection. The weekly increase of approximately 40 g is equivalent to the haemoglobin contained in one bag of 450 mL stored blood [43]. The delay of approximately one week between rHuEpo injections and the observed responses in Hb_mass_ and red cell volume is explained by the reticulocyte maturation time of 1–4 days [44]. While normal human red blood cells can theoretically persist in the circulation for approximately 17 weeks [5], Hb_mass_ and red cell volume started to decrease toward baseline values from approximately the second week post rHuEpo administration with a similar rate to that of the increase during administration. This decrease in Hb_mass_ and red cell volume post rHuEpo administration entailed the haemolysis of circulating red blood cells and therefore the release of heme from heamoglobin [45]. The heme degradation is catalyzed by heme oxygenase and generates one molecule of CO per molecule of heme oxidized [46]. The CO produced by heme metabolism represents more than 85% of the endogenous CO production in healthy non-smoking subjects [46]. Although measurement of blood carboxyhaemoglobin is influenced by environmental factors such as air pollution [5], carboxyhaemoglobin can be used as an index of haemolysis [47]. Indeed, basal carboxyhaemoglobin levels increased at the end of the rHuEpo administration and remained elevated for 2 weeks post administration reflecting accelerated haemolysis. Despite being only an indirect marker, these results are in agreement with the negative regulation of red cell mass by neocytolysis when supraphysiologic red cell mass and endogenous erythropoietin suppression occur (induced by rHuEpo administration, for instance) [7], [8]. In addition, this study confirmed that 4 weeks of rHuEpo administration sufficient to induce a significant increase in haematocrit did not increase resting blood pressure in healthy subjects when blood pressure was assessed by standard techniques compared to intra-arterial pressure transducers [40]. This study also demonstrates that the optimized CO-rebreathing method performed weekly for ten weeks is safe and well tolerated by healthy subjects.

### Plasma Volume

Apart from confirming previous observations that rHuEpo administration increases haemoglobin concentration by increasing Hb_mass_ and by decreasing plasma volume [2], [3], the present study showed that plasma volume returned to pre-injection values only approximately 4 weeks post rHuEpo administration. Previous studies have concluded that plasma volume returned rather rapidly toward baseline value thus restoring pre-administration haemoglobin concentration values without affecting red cell volume [3], [4]. Olsen et al. [3] used injections of 5000 IU rHuEpo administered every second day for 2 weeks and thereafter only once a week for the 2 consecutive weeks to demonstrate that rHuEpo down-regulates the renin-angiotensin-aldosterone system as well as the rate of proximal renal tubular reabsorption and glomerular filtration. The longer and more contrasted rHuEpo administration regimen used in the present study with a more abrupt erythropoiesis stimulation termination may have induced a more profound down-regulation of the renin-angiotensin-aldosterone system and therefore, may explain why plasma volume had not been restored until 4 weeks after rHuEpo administration. In agreement with previous findings which used a similar rHuEpo regimen than the present study, pre-injection haemoglobin concentration values were not restored 4 weeks post administration [28].

In conclusion, relative to baseline, running performance was significantly improved following 4 weeks of rHuEpo administration in trained men and remained significantly elevated 4 weeks after administration by approximately 6% and 3%, respectively. These performance effects coincided with significantly rHuEpo-induced elevated 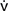 O_2 max_ and Hb_mass_.
